# Limiting Monoamines Degradation Increases L-DOPA Pro-Locomotor Action in Newborn Rats

**DOI:** 10.3390/ijms241914747

**Published:** 2023-09-29

**Authors:** Inès Khsime, Marie Boulain, Abderrahman Fettah, Abdeslam Chagraoui, Gilles Courtand, Philippe De Deurwaerdère, Laurent Juvin, Grégory Barrière

**Affiliations:** 1 Univ. Bordeaux, CNRS, INCIA, UMR5287, F-33000 Bordeaux, Franceabderrahman.fettah@u-bordeaux.fr (A.F.); gilles.courtand@u-bordeaux.fr (G.C.); laurent.juvin@u-bordeaux.fr (L.J.); 2 Laboratory of Neuronal and Neuroendocrine Differentiation and Communication, UNIROUEN, INSERM U1239, Institute for Research and Innovation in Biomedicine of Normandy (IRIB), F-76000 Rouen, France; abdeslam.chagraoui@univ-rouen.fr; 3 Department of Medical Biochemistry, Rouen University Hospital, CHU de Rouen, F-76000 Rouen, France

**Keywords:** air-stepping, monoamines, L-DOPA, spinal cord, newborn rat

## Abstract

L-DOPA, the precursor of catecholamines, exerts a pro-locomotor action in several vertebrate species, including newborn rats. Here, we tested the hypothesis that decreasing the degradation of monoamines can promote the pro-locomotor action of a low, subthreshold dose of L-DOPA in five-day-old rats. The activity of the degrading pathways involving monoamine oxidases or catechol-O-methyltransferase was impaired by injecting nialamide or tolcapone, respectively. At this early post-natal stage, the capacity of the drugs to trigger locomotion was investigated by monitoring the air-stepping activity expressed by the animals suspended in a harness above the ground. We show that nialamide (100 mg/kg) or tolcapone (100 mg/kg), without effect on their own promotes maximal expression of air-stepping sequences in the presence of a sub-effective dose of L-DOPA (25 mg/kg). Tissue measurements of monoamines (dopamine, noradrenaline, serotonin and some of their metabolites) in the cervical and lumbar spinal cord confirmed the regional efficacy of each inhibitor toward their respective enzyme. Our experiments support the idea that the raise of monoamines boost L-DOPA’s locomotor action. Considering that both inhibitors differently altered the spinal monoamines levels in response to L-DOPA, our data also suggest that maximal locomotor response can be reached with different monoamines environment.

## 1. Introduction

L-DOPA is a medication that has been approved for the treatment of several diseases including some forms of dystonia and, of course, Parkinson’s disease. Other medical applications, such as the locomotor impairments consequent to spinal cord injuries, could benefit from L-DOPA injection, but it would need a better understanding of its mechanisms of action in the spinal cord. Indeed, the therapeutic benefit of L-DOPA is much more complex than an increase in dopaminergic transmission associated with the decarboxylation of L-DOPA in dopaminergic terminals. Actually, some of the effects of L-DOPA can be independent of dopamine itself [1,2,3,4], and because the modulatory role of monoamines in the effects of L-DOPA is not always obvious, it usually requires deeper pharmacological analysis.

From the initial studies of the late 60 s, it is well established that L-DOPA administration triggers locomotor activities in cats and rabbits [5,6,7,8,9,10,11,12]. Since then, L-DOPA proved to be efficient in triggering locomotor episodes in a variety of vertebrate species, preparations and conditions [13,14,15,16]. The newborn rat is an interesting model for addressing the locomotor effects of L-DOPA since its peripheral administration triggers episodes of air-stepping in a dose-dependent manner [17,18]. Combining kinematic and biochemical approaches, we recently showed that the pro-locomotor action of L-DOPA was paralleled by an accumulation of L-DOPA itself and its by-end product dopamine in the spinal cord [19]. The aromatic amino-acid decarboxylase (AADC), which is responsible for the transformation of L-DOPA into dopamine, is expressed in the spinal cord in the perinatal period [20,21]. Thus, the pro-locomotor action of L-DOPA could be in part mediated through its intraspinal production of dopamine.

In our previous study, we showed that L-DOPA administered peripherally was not effective at the dose of 25 mg/kg to trigger air-stepping in the newborn rat. The threshold to trigger the behavior in all animals is around 50 mg/kg, the most robust episodes being obtained at even higher doses (75–100 mg/kg [19]). In a context where one would use L-DOPA administration as a support to facilitate the expression of locomotor activity in pathological contexts, such as following a spinal cord injury, chronic or repeated L-DOPA administration at high dose can be a burden [1]. In this context, designing combinatorial pharmacological approaches based on low doses of L-DOPA to trigger locomotor behavior can be a suitable alternative.

Initial studies carried out in cats and rabbits combined the administration of L-DOPA with niamide (also known as nialamide), which is a monoamine oxidase inhibitor (MAOi). By inhibiting the action of MAOs (MAO A and MAO B) peripherally and centrally, this drug is likely to increase the central levels of monoamines including dopamine, noradrenaline and serotonin (5-HT). The catechol-O-methyl transferase is another enzyme working in parallel with MAOs in the metabolism of L-DOPA and catecholamines [1,22]. The COMT inhibitor (COMTi) tolcapone has been used in Parkinson’s disease to enhance the penetration of L-DOPA in the brain and ameliorate the behavioral responses [23]. Therefore, inhibiting the activity of the COMT should also increase the peripheral and central levels of L-DOPA and catecholamines, but with a limited action on 5-HT. Thus, combining a low dose of L-DOPA with nialamide or tolcapone could be a relevant pharmacological approach to trigger air-stepping in newborn rat. 

Here, we tested the following hypothesis: combining a low (sub-threshold) dose of L-DOPA with either the MAOi nialamide or the COMTi tolcapone is efficient in triggering locomotor episodes in newborn rats, assuming that both inhibitors contribute to increasing the central levels of monoamines. For this purpose, we first measured the expression of air-stepping activities in different groups of animals receiving one of these cocktails, and immediately after quantified the spinal content of L-DOPA and monoamines in the cervical and lumbar segments where the locomotor networks are located. These effects were compared to a calibration of the effects of L-DOPA administrated alone at high doses.

## 2. Results

**Figure 1:**
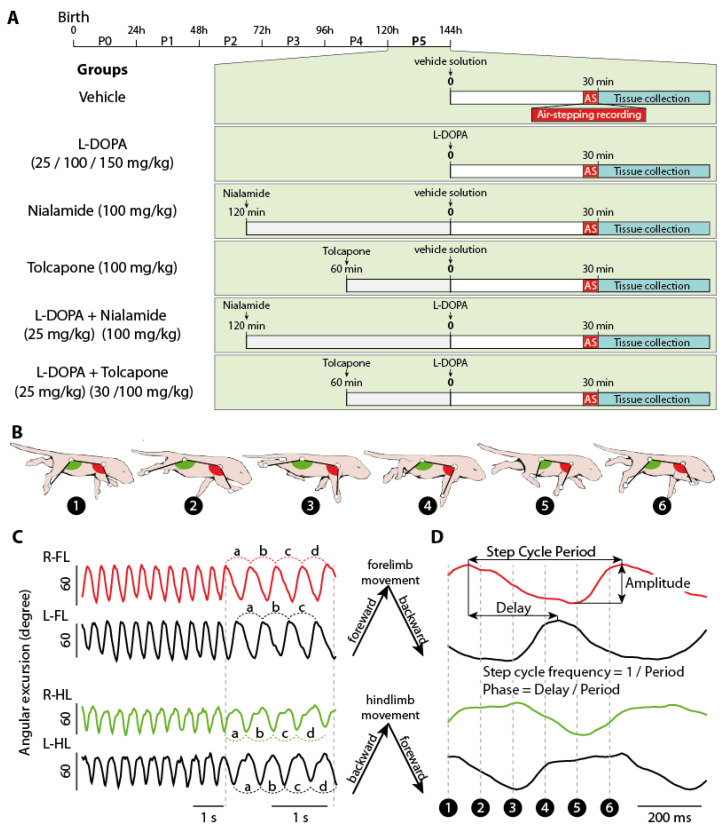
Experimental paradigm. (**A**) Timeline of the experiments and experimental groups. (**B**) Schematic diagrams of a newborn rat expressing air-stepping. Each diagram represents the animal at a different timepoint of a step cycle. The angular excursion of the limbs are measured from the kinematic markers (white dots). (**C**) Angular excursions of all 4 limbs during a sequence of air-stepping. On the enlarged portion (middle) are shown 4 successive step cycles (a, b, c, d) together with the direction of the forelimb and hindlimb movements. (**D**) Angular excursions of one single step cycle from which were calculated the different locomotor parameters. Dotted lines indicate the timepoints corresponding to the schematic diagrams in (**B**).

The capacity of L-DOPA to trigger episodes of air-stepping when administrated alone or following a pre-injection of either tolcapone (COMTi, 1 h prior L-DOPA) or nialamide (MAOi, 2 h prior L-DOPA) was studied in five-day-old rats. In order to establish a possible link between the locomotor activity and the monoaminergic neuromodulatory environment in the vicinity of the spinal pattern generators of locomotion, the cervical and lumbar segment were dissected out for dosage purposes 30 min after L-DOPA administration when a peak of action is reached [6] (in-house verification). Accordingly, the locomotor behavior expressed during the last 3 min prior to tissue collection was analyzed. The time frame of our experiments and the different experimental groups are presented in Figure 1A.

### 2.1. Nialamide and Tolcapone Potentiate the Pro-Locomotor Action of L-DOPA

**Figure 2:**
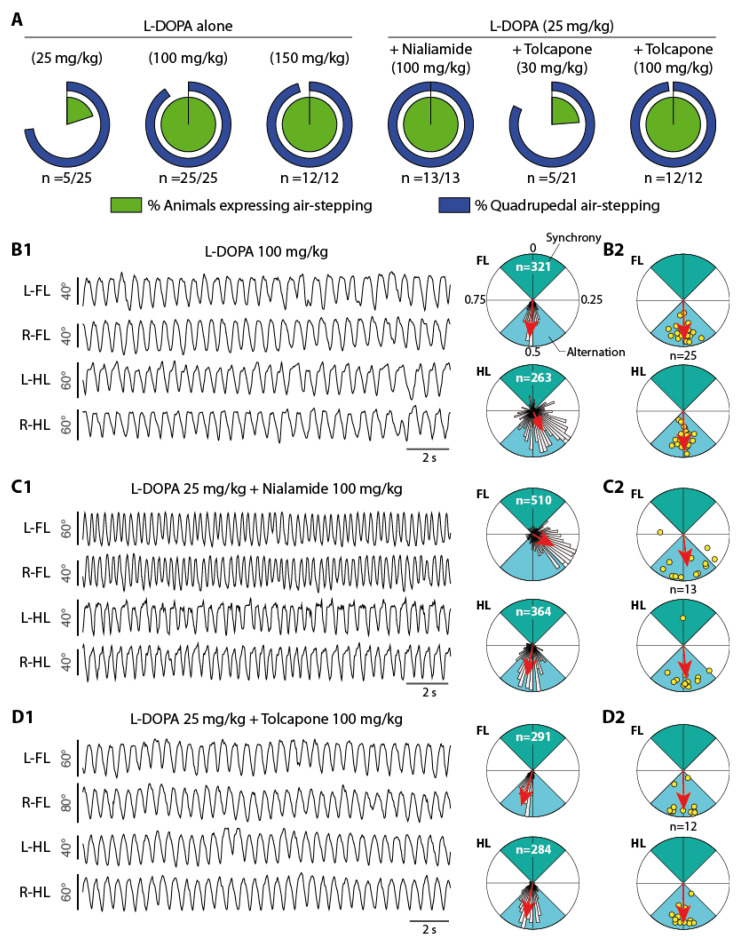
L-DOPA-based strategies to trigger air-stepping in rat pups. (**A**) Percentages of animals expressing air-stepping movements (green diagram) and of quadrupedal air-stepping activity (blue diagram) in all L-DOPA-based pharmacological conditions. (**B1–D1**) Examples of air-stepping activity obtained from three different animals that received L-DOPA at the dose of 100 mg/kg (**B1**), nialamide 100 mg/kg + L-DOPA 25 mg/kg (**C1**) and tolcapone 100 mg/kg + L-DOPA 25 mg/kg (**D1**). The sequence are presented as angular excursions of all 4 limbs around the scapula (forelimbs) and hip (hindlimbs) joints. Polar plots (circular statistics) on the right illustrate the phase relationship between the locomotor movements of the bilateral forelimbs (upper plots) and hindlimbs (bottom plots) measured from each example. The direction and length of each red arrow indicate the mean phase value and associated r value, respectively. The distribution of raw values (which number is indicated in white) are also provided as histograms using bins of 5°. The green and blue quadrants illustrate the portions corresponding to synchrony and alternation, respectively. (**B2–D2**) Polar plots (circular statistics) of the mean phase relationships measured in all animals tested in each of the three pharmacological conditions. Each animal is represented by a yellow dot placed at the mean direction and r distance from the origin. The direction and length of each red arrow indicate the mean phase and associated r value calculated from all animals (number of animals in each group are indicated in black between the diagrams). L/R: left / right, FL/HL: Forelimb / Hindlimb.

All animals of the vehicle group (*n* = 13) remained motionless during the 30 min period of investigation. Moreover, L-DOPA injected at a low dose of 25 mg/kg was ineffective to consistently trigger a locomotor activity since only 5 of 25 animals (20%) demonstrated air-stepping movements (Figure 2A). This is in contrast with the robust episodes of air-stepping that were systematically observed in animals receiving the dose of 100 (*n* = 25/25) or 150 mg/kg L-DOPA (*n* = 12/12, Figure 2A). At these doses, animals expressed a locomotor activity almost continuously with a percentage of quadrupedal locomotion reaching 90 ± 9 % (100 mg/kg) to 95 ± 7% (150 mg/kg, Figure 2A).

It is of interest that a low dose of 25 mg/kg L-DOPA became effective to trigger air-stepping when administrated 2 h after a pre-injection of nialamide (MAOi, 100 mg/kg). In this experimental condition (*n* = 13), all animals expressed robust, continuous, sequences of air-stepping (Figure 2A). Furthermore, it is important to note that nialamide alone (100 mg/kg) was ineffective in triggering any locomotor activity in all tested pups (*n* = 10).

The results obtained with tolcapone (COMTi) administered 1 h before L-DOPA differed as a function of the dose used. At the dose of 30 mg/kg, tolcapone did not increase the probability of obtaining air-stepping in pups receiving 25 mg/kg L-DOPA since only 5 out of 21 animals in this experimental group expressed a locomotor activity. Overall, the activity profile in this experimental group was similar to the one obtained in the group of animals receiving L-DOPA (25 mg/kg) alone (Figure 2A). However, increasing the dose of tolcapone to 100 mg/kg invariably increased the potency of 25 mg/kg L-DOPA to trigger air-stepping. All animals consistently walked in the air virtually the whole investigation (*n* = 12/12). This contrasted the complete absence of activity observed in all animals receiving tolcapone (100 mg/kg) without L-DOPA (*n* = 10). We did not look at the effect of tolcapone (30 mg/kg) alone on air-stepping, but it was totally ineffective in producing movements before the injection of L-DOPA (25 mg/kg).

### 2.2. Modulation of the Locomotor Parameters

We next compared the locomotor parameters measured between the different groups. In this context, we did not include the data obtained in the group L-DOPA (25 mg/kg) alone nor those from the group tolcapone (30 mg/kg)/L-DOPA (25 mg/kg) because of the few numbers of animals stepping in these conditions and the lack of permissive action of tolcapone at this dose.

A fragment of air-stepping sequence recorded from an animal receiving a dose of 100 mg/kg L-DOPA is presented in Figure 2(B1). In this representation, the pendular movements of bilateral hind- and forelimbs are represented as angular variations over time around the hip and scapular axes, respectively. It is noteworthy that air-stepping activities were dominated by the expression of locomotor movements that alternated bilaterally both at the fore- and hindlimb levels. This is illustrated on the polar plots (Figure 2(B1), right panel) where the left/right coupling values measured between pairs of limbs are pooled as circular histograms (binning 5°) together with the mean direction (red arrows). The concentration of values pointing in the bottom direction (toward a value of 0.5, blue sector) are indicative of a predominant alternating activity. As illustrated from the polar plots summarizing the data obtained at the scale of this experimental group (*n* = 25, Figure 2(B2)), alternating locomotor movements are a typical feature of air-stepping induced by L-DOPA 100 mg/kg. It is of interest that alternating movements also predominated during the sequences of air-stepping induced by combining 25 mg/kg L-DOPA with one of the MAOi (Figure 2(C1,C2)) or COMTi (Figure 2(D1,D2)).

**Figure 3:**
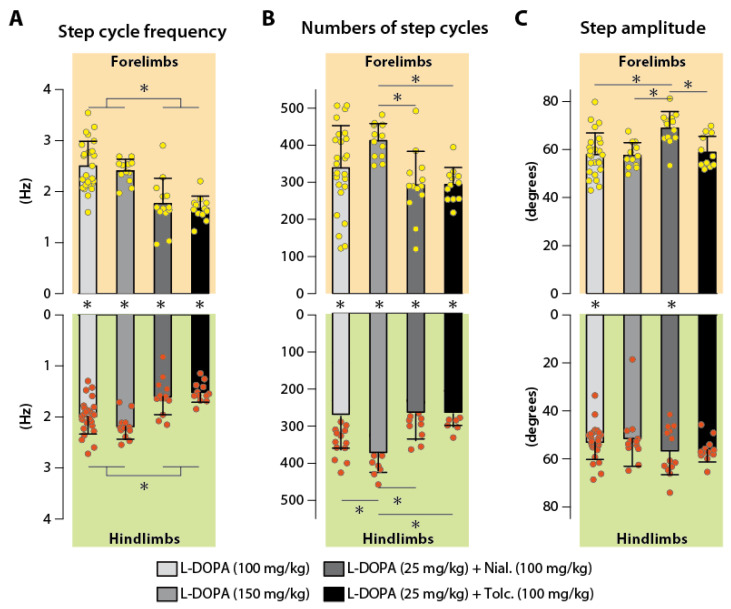
Locomotor parameters measured in response to the most efficient pro-locomotor pharmacological treatments. Histograms of the number of step cycles (**A**), step cycle frequency (**B**) and locomotor movement amplitude (**C**) obtained in the groups of animals expressing air-stepping in response to L-DOPA 100 and 150 mg/kg, and L-DOPA 25 mg/kg associated with either nialamide or tolcapone at 100 mg/kg. Forelimb/Hindlimb differences in each pharmacological conditions are indicated by asterisks placed between the horizontal histograms. Asterisks placed at the extremity of the histograms highlight the statistical differences obtained between the pharmacological groups at the forelimb and hindlimb levels (statistics in the Section 2). Yellow and red dots represent individual values obtained at the forelimb an hindlimb levels, respectively.

However, at this early stage of development, pups generally do not maintain a 1:1 coordination between the fore- and hindlimb. This is notably exemplified on Figure 2(C1) for one animal receiving nialamide (MAOi) and L-DOPA and which manifested forelimb locomotor movements at a higher rate compared to the hindlimbs. Overall, comparing the data obtained in all experimental groups showed that none of the pharmacological conditions investigated here allowed for the homogenization of the forelimb and hindlimb stepping mean frequencies that remained always higher at the level of the scapular girdle (Figure 3A, paired *t*-test: L-DOPA 100 and 150, *p* < 0.0001; L-DOPA 25/nialamide, *p* = 0.0002; L-DOPA 25/tolcapone, *p* = 0.0014). We also noticed that the stepping frequencies obtained with L-DOPA (100 and 150 mg/kg) were significantly higher compared to the ones measured in the groups of animals receiving nialamide or tolcapone with L-DOPA (25 mg/kg, Figure 3B, ANOVA with Tukey’s multiple comparisons: forelimb, F(3,58) = 16.7, *p* < 0.0001; hindlimb, F(3,58) = 14.38, *p* < 0.0001).

In addition, we found that the number of steps was higher at the forelimb level whatever the nature of the pharmacological treatment (Figure 3A, Paired *t*-test: L-DOPA 100 and 150, *p* < 0.0001; L-DOPA 25/nialamide, *p* = 0.0180; L-DOPA 25/tolcapone, *p* = 0.0017). However, the number of steps was not significantly different between the groups L-DOPA (100 mg/kg), and L-DOPA (25 mg/kg) combined with either tolcapone or nialamide. It is of note that the highest number of steps were observed in the group of rats receiving L-DOPA at the highest dose of 150 mg/kg (ANOVA with Tukey’s multiple comparisons: Forelimb, F(3,58) = 4.866, *p* = 0.0044; Hindlimb, F(3,58) = 4.866, *p*= 0.0005).

Finally, we found that all pharmacological treatments triggered hindlimb stepping movements of similar amplitude (Figure 3C, ANOVA: F(3,58) = 1.154, *p* = 0.3351). At the level of the forelimbs, the amplitude of the stepping movements was slightly but significantly higher in the L-DOPA with nialamide condition compared to the other pharmacological treatments (ANOVA with Tukey’s multiple comparisons, F(3,58) = 7.127, *p* = 0.0004).

### 2.3. Biochemical Correlates

Immediately after the stepping session, the spinal tissue was collected in order to measure the contents of L-DOPA, monoamines and some of their metabolites in the cervical and lumbar spinal cord using high performance liquid chromatography (HPLC).

#### 2.3.1. Basal Monoamines Levels

**Figure 4:**
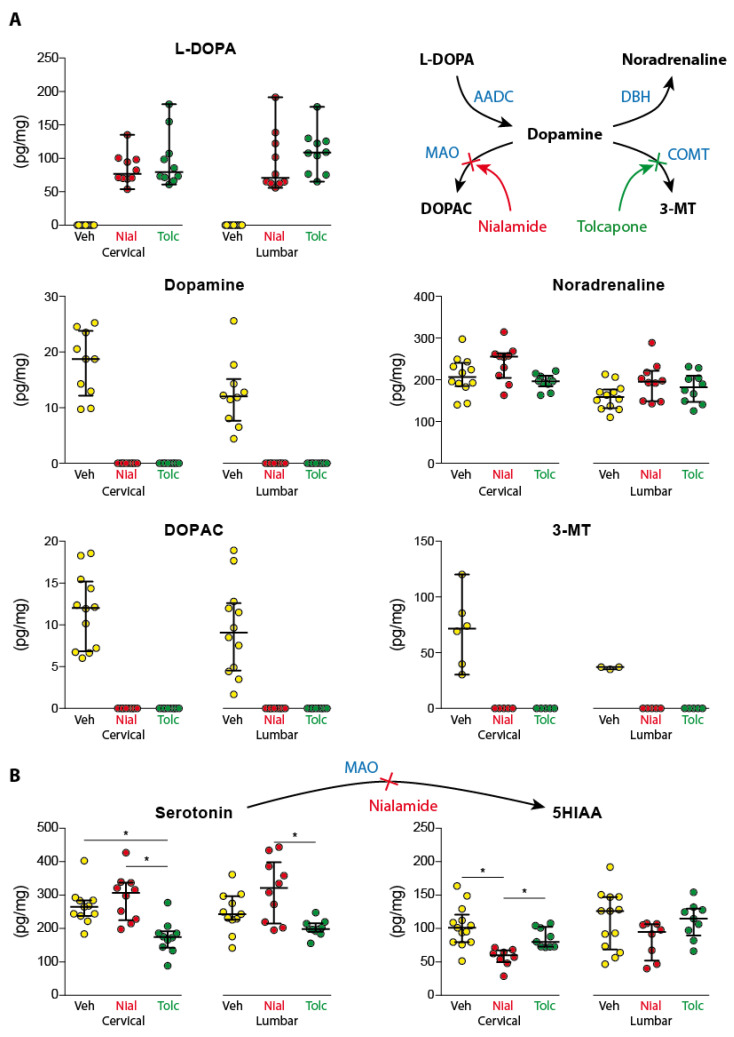
Spinal content of monoamines and associated by-products following the administration of nialamide or tolcapone at the dose of 100 mg/kg. (**A**) Both drugs exert the same effect on the basal levels of catecholamines in the cervical and lumbar spinal cord. Both reveal the presence of endogenous L-DOPA and devrease the dopamine, DOPAC and 3-MT to levels below detection threshold. No effect is observed on noradrenaline content. (**B**) Nialamide slightly increases spinal levels of serotonin while decreasing the basal level of its metabolite 5HIAA. AADC: Aromatic amino-acid decarboxylase; DBH: Dopamine-β-hydroxylase; MAO: Monoamine oxidase; COMT: Catechol-O-methyltransferase; DOPAC: 3,4-Dihydroxyphenylacetix acid; 3-MT: 3-Methoxytyramine; 5HIAA: 5-Hydroxyindolacetic acid. * *p* < 0.05.

L-DOPA in the cervical and lumbar spinal cord remained below its detection threshold in all animals receiving the vehicle solution (*n* = 13). In contrast, dopamine (*n* = 10/13), DOPAC (3,4-Dihydroxyphenylacetic acid) and noradrenaline (*n* = 12/13), 5-HT (*n* = 11/13 and 5-HIAA (*n* = 13/13) could be reliably measured at both cervical and lumbar spinal levels (Figure 4A, Table 1). Paired comparisons (Table 1) revealed no significant regional differences for compounds at the only exception of noradrenaline that was found in greater amount in the cervical cord.

No DOPAC was detected in the spinal cord in the group of animals receiving nialamide (100 mg/kg, *n* = 10), as expected from its inhibitory action on the activity of the monoamine oxidases. Unexpectedly, dopamine was not detected in the cervical and lumbar cord, as 3-Methoxytyramine (3-MT). Interestingly, in the group receiving tolcapone alone (100 mg/kg, *n* = 10) no 3-MT, no dopamine and no DOPAC were detected in the spinal cord. In contrast, endogenous L-DOPA could be detected after nialamide or tolcapone (Figure 4A, Table 1) in the cervical and lumbar cord. The levels of endogenous L-DOPA were not different between the nialamide and tolcapone groups (Mann–Whitney, cervical *p* value = 0.4727; lumbar *p* value = 0.1485). Cervical (Kruskal-Wallis, *p* value = 0.0563) and lumbar (Kruskal–Wallis, *p* value = 0.1501) noradrenaline levels measured in the vehicle, tolcapone and nialamide groups were not different.

In contrast, the levels of 5-HT were affected by the treatment in the cervical (Kruskal–Wallis, *p* value = 0.0005) and lumbar cord (Kruskal–Wallis, *p* value = 0.0069). Multiple comparisons showed that 5-HT levels were significantly lower in the group tolcapone compared to the vehicle (*p* value = 0.0095) and nialamide (*p* value = 0.0007) groups. The same observation was made at the lumbar levels where the difference reached significance between the tolcapone and nialamide groups (*p* value = 0.0054). In all experimental conditions, however, no regional differences between the cervical and lumbar 5-HT levels were found (Figure 4B, Table 1). 5-Hydroxyindolacetic acid (5-HIAA) is a two-step by-product of 5-HT involving the MAO. Our data showed that the 5-HIAA levels depended also on the treatment in the cervical (Kruskal–Wallis, *p* value = 0.0003) but not in the lumbar cord (Kruskal–Wallis, *p* value = 0.1831). At the cervical level, multiple comparisons showed that nialamide significantly decreased the level of 5-HIAA compared to the saline (*p* value = 0.0002) and tolcapone (*p* value = 0.0228) groups.

#### 2.3.2. L-DOPA at Different Doses

**Figure 5:**
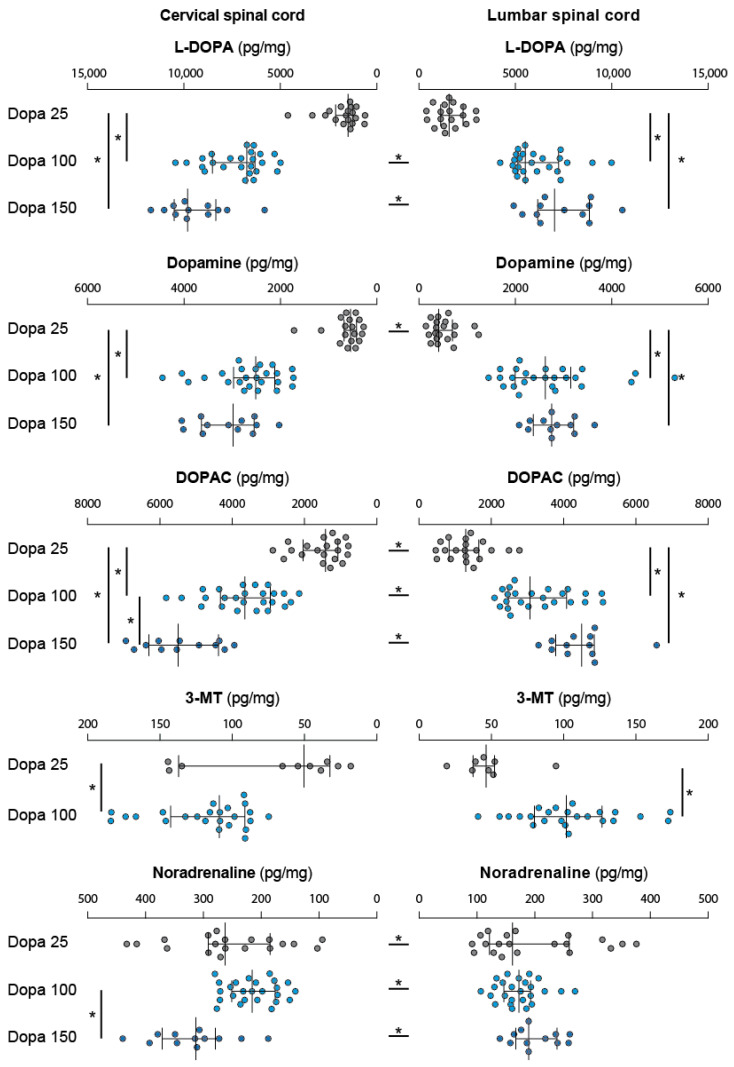
Spinal catecholamines levels in response to administration of low and high doses of L-DOPA. Increasing the dose of L-DOPA from the low dose of 25 mg/kg to the high doses of 100 and 150 mg/kg overall affect the catecholamine content similarly in the cervical (left panels) and lumbar cord (right panels). At the exception of noradrenaline which remain mostly unaffected, L-DOPA, dopamine, DOPAC and 3-MT were increased from low to the highest dose of injected L-DOPA. Forelimb/hindlimb differences in each pharmacological conditions are indicated by asterisks placed between the horizontal histograms. Asterisks placed at the extremity of the histograms highlight the statistical differences obtained between the pharmacological groups at the forelimb and hindlimb levels. DOPAC: 3,4-Dihydroxyphenylacetix acid; 3-MT: 3-Methoxytyramine.

In agreement with our recent work [19], measured L-DOPA, which remained below its detection threshold following saline injection (see above), uniformly accumulated in the cervical and lumbar enlargements 30 min after its administration at the dose of 25 mg/kg (Figure 5, Table 1). It is noteworthy that the tissue levels of L-DOPA measured after 25 mg/kg L-DOPA (Figure 5) are approximately 10 to 20-fold higher compared to the levels measured after nialamide or tolcapone alone (Figure 4A, Table 1). Increasing the dose from 25 to 100 and 150 mg/kg increased the tissue concentration of L-DOPA at both spinal cord levels (Figure 5, Table 2). Multiple comparisons showed that the spinal content of L-DOPA plateaued since no significant difference could be established between the doses of 100 and 150 mg/kg in the cervical and lumbar segments (Figure 5, Table 2).

L-DOPA at 25 mg/kg significantly increased the spinal content of dopamine in the cervical (Vvhicle median/IQR: 0.0187/0.0116 ng/mg, L-DOPA median/IQR: 0.5466/0.2564 ng/mg, Mann–Whitney, *p* < 0.0001) and lumbar (vehicle median/IQR: 0.012/0.0074 ng/mg, L-DOPA median/IQR = 0.4119/0.3081 ng/g, Mann–Whitney, *p* < 0.0001) enlargements compared with the basal, low levels measured in vehicle-treated pups. Although close, the amount of dopamine was significantly higher in the cervical region (Figure 5, Table 1). Because the low dopamine levels measured in vehicle condition biased the multiple comparisons, this condition was excluded. We found that the spinal levels of dopamine were significantly raised in the cervical and lumbar regions following administrations of L-DOPA at 100 and 150 mg/kg (Figure 5, Table 2).

As expected, the levels of DOPAC were also increased in the cervical and lumbar cord (Table 2). The results obtained for 3-MT are scarcer since we transiently lost the capacity to reliably measure its levels during the course of these experiments (Figure 5). Data that were collected with confidence in the groups receiving L-DOPA 25 mg/kg (*n* = 10) and 100 mg/kg (*n* = 24) indicated that the levels of 3-MT increased with the dose of L-DOPA in the cervical (Mann–Whitney, *p* = 0.0046) and lumbar spinal cord (Mann–Whitney, *p* < 0.0001, Figure 5). The dose of L-DOPA did not impact the levels of noradrenaline across groups in the lumbar spinal cord in contrast to the cervical cord where the multiple comparisons highlighted higher NA levels in the group L-DOPA 150 mg/kg compared to the group L-DOPA 100 mg/kg (Table 2).

**Figure 6:**
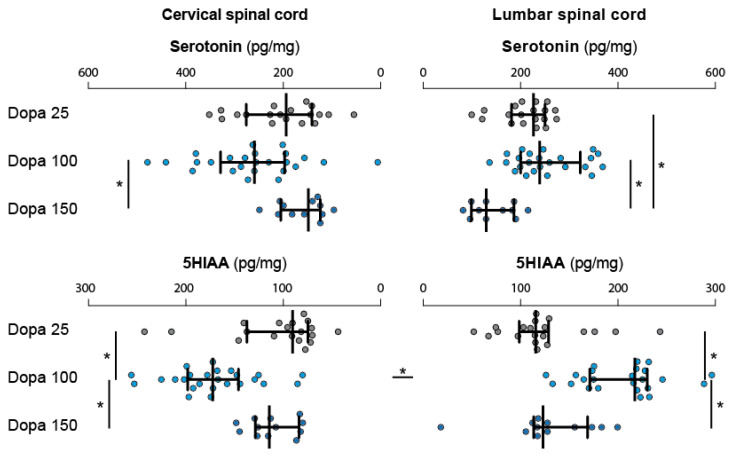
Spinal serotonin levels in response to the administration of low and high doses of L-DOPA. L-DOPA injection is likely to affect moderately but significantly the spinal serotonergic system, the main effect being observed with the dose of 100 mg/kg at which the cervical and lumbar 5HIAA levels are increased. Forelimb/hindlimb differences in each pharmacological conditions are indicated by asterisks placed between the horizontal histograms. Asterisks placed at the extremity of the histograms highlight the statistical differences obtained between the pharmacological groups at the forelimb and hindlimb levels. 5HIAA: 5-Hydroxyindolactic acid.

Spinal levels of 5-HT were also affected by the dose of L-DOPA. In the cervical cord, 5-HT levels were significantly lower in the group L-DOPA 150 mg/kg compared to the group L-DOPA 100 mg/kg (Figure 6, Table 2). The same applied to the lumbar cord where 5-HT levels were significantly lower in the group L-DOPA 150 mg/kg compared to the groups L-DOPA 25 and 100 mg/kg (Table 2). In the same groups, 5-HIAA levels were significantly higher in the group L-DOPA 100 mg/kg compared to the L-DOPA 25 and 150 mg/kg at both spinal cord levels (Figure 6, Table 2). It is of note that no differences were found between the cervical and lumbar 5-HT levels (Table 1).

#### 2.3.3. L-DOPA with Nialamide or Tolcapone

**Figure 7:**
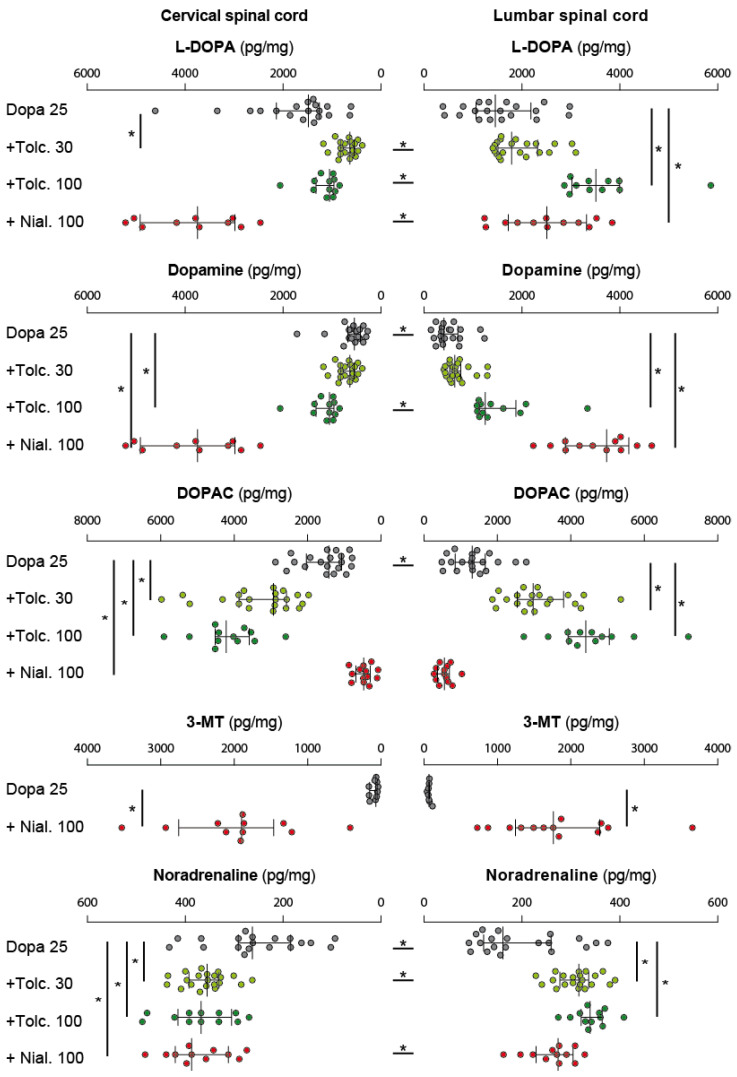
Nialamide and tolcapone differently modulate the catecholamine spinal levels in response to administration of low L-DOPA dose. Note that the main difference between tolcapone and nialamide is the capacity of the later to highly increase the spinal content of L-DOPA and dopamine. As expected, the levels of DOPAC and 3-MT measured in the presence of tolcapone and nialamide validate their respective inhibitory actions towards the COMT and MAOs enzymes, respectively. Forelimb/hindlimb differences in each pharmacological conditions are indicated by asterisks placed between the horizontal histograms. Asterisks placed at the extremity of the histograms highlight the statistical differences obtained between the pharmacological groups at the forelimb and hindlimb levels.

Finally, we analyzed the biochemical consequences of pre-administrating the MAOi nialamide or the COMTi tolcapone before injecting L-DOPA at the low dose of 25 mg/kg. In comparison with the situation where L-DOPA is injected alone, nialamide pre-treatment increased the spinal content of L-DOPA, especially in the cervical cord where higher levels were found compared to the lumbar area (Figure 7, Table 3).

Used as a cocktail with L-DOPA (25 mg/kg), tolcapone at a dose of 30 mg/kg decreased the level of L-DOPA measured in the cervical cord in comparison to the group receiving L-DOPA only (Figure 7, Table 3). This contrasts with the lumbar cord where the levels of L-DOPA were not statistically different between the groups receiving either L-DOPA alone or combined with tolcapone 30 mg/kg (Figure 7, Table 3). Increasing the dose of tolcapone to 100 mg/kg led to an increase of L-DOPA content in lumbar cord as compared with the group receiving L-DOPA only (Figure 7). When compared with the group of animals receiving L-DOPA alone, pretreatment with tolcapone (100 mg/kg) allowed keeping unaltered the cervical content of L-DOPA while significantly increasing its lumbar levels (Figure 7, Table 3).

Tolcapone, administered at 30 mg/kg before L-DOPA 25 mg/kg, did not induce any effect on the spinal accumulation of dopamine in response to L-DOPA (Figure 7, Table 3). Only the pre-treatment by 100 mg/kg tolcapone increased moderately but significantly the amount of lumbar dopamine induced by L-DOPA (25 mg/kg) (Figure 7, Table 3). The highest spinal levels of dopamine were obtained when the MAOi nialamide 100 mg/kg was associated with 25 mg/kg L-DOPA (Figure 7, Table 3). Combining nialamide with 25 mg/kg L-DOPA allowed further increasing dopamine levels in the lumbar cord specifically compared to the group receiving the highest dose of L-DOPA (150 mg/kg, unpaired *t*-test, *p* = 0.0052). Finally, in most pharmacological conditions, the levels of dopamine were not different between the cervical and lumbar enlargements (Figure 7, Table 1).

Nialamide induced an overall reduction of DOPAC levels in the spinal cord in response to L-DOPA (25 mg/kg), especially in the cervical region where the difference reached significance (Figure 7, Table 3). In contrast, tolcapone (30 and 100 mg/kg) further increased DOPAC levels induced by L-DOPA in the cervical and lumbar spinal cord (Figure 7, Table 3). As expected, the COMTi (30–100 mg/kg) suppressed the increase in 3-MT induced by L-DOPA. Contrariwise, 3-MT levels were significantly increased when nialamide (100 mg/kg) was given with 25 mg/kg L-DOPA (Figure 7). Therefore, tolcapone and nialamide pre-treatments exert opposite actions on the spinal level of DOPAC and 3-MT measured following L-DOPA administration.

Overall, we found an effect of the pharmacological cocktails on the levels of noradrenaline both in the cervical and lumbar spinal segments. As expected, noradrenaline levels were significantly elevated in the groups of animals that received either tolcapone or nialamide prior to L-DOPA (Figure 7, Table 3). Finally, we noticed that, in virtually all experimental groups, the levels of noradrenaline were significantly higher in the cervical than in the lumbar enlargement (Table 1).

**Figure 8:**
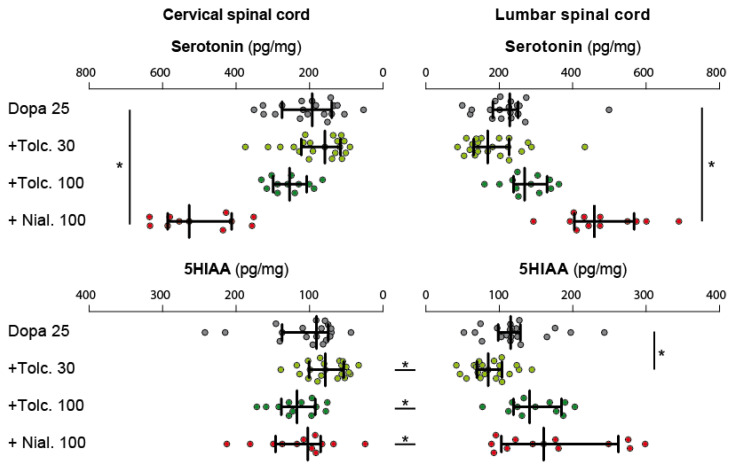
Nialamide but not tolcapone increases the serotonin level measured in response to low L-DOPA dose. Forelimb/hindlimb differences in each pharmacological conditions are indicated by asterisks placed between the horizontal histograms. Asterisks placed at the extremity of the histograms highlight the statistical differences obtained between the pharmacological groups at the forelimb and hindlimb levels.

Figure 8 illustrates the levels of 5-HT measured in the cervical and lumbar regions of the spinal cord in all experimental groups. Pairwise comparisons between the cervical and lumbar 5-HT levels showed no statistical differences between the two spinal cord regions in all experimental conditions (Table 1). Using the group receiving L-DOPA 25 mg/kg as a reference for the multiple comparisons, we found that 5-HT levels were stable across the experimental conditions at both spinal cord levels (Table 3). Only the group of animals receiving nialamide (100 mg/kg) and L-DOPA (25 mg/kg) demonstrated higher levels of 5-HT that reached statistical significance. In contrast, we found a regional difference in the level of 5-HIAA in most pharmacological conditions. In all cases, the levels of 5-HIAA were higher in the lumbar cord compared to the cervical enlargement (Table 1). Overall, the spinal levels of 5-HIAA measured in the different pharmacological conditions were not significantly different from the one measured in the group L-DOPA (25 mg/kg), taken here as reference (Table 3).

### 2.4. Correlations

**Figure 9:**
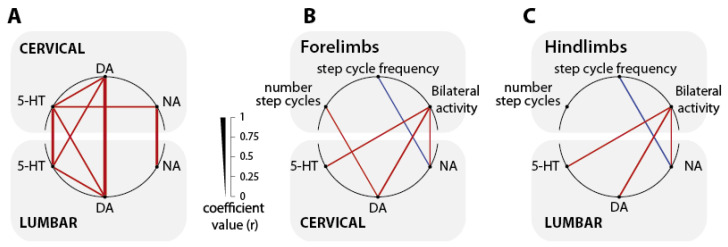
Correlations between spinal monoamines levels and the locomotor parameters. Schematic diagrams illustrating the correlations between the lumbar and cervical spinal cord monoamines (**A**), between the cervical monoamines levels and the forelimb locomotor parameters (**B**), and between the lumbar monoamines levels and the hindlimb locomotor parameters (**C**). Line thickness is proportional to the correlation coefficients (r) according to the scale presented on the right of panel A. Only statistically significant correlations are indicated. Positive and negative correlations are indicated in red and blue, respectively. 5-HT: Serotonin; DA: Dopamine; NA: Noradrenaline.

Finally, we aimed at correlating the behavioral and biochemical data in considering the animals that received L-DOPA 25 mg/kg alone or with nialamide or tolcapone. We first tested the correlations between the levels of monoamines measured in the cervical and lumbar cord (Figure 9A, [24]). Each monoamine level measured in the lumbar cord correlated positively to the one measured in the cervical cord. Next, these analyses also revealed a consistent positive correlation between the dopamine and serotonin levels in the lumbar and cervical cord. Interestingly, no correlations were found between dopamine and noradrenaline. We next addressed from the animals that expressed a locomotor activity the correlations between the locomotor parameters and the levels of monoamines (Figure 9B,C). Positive correlations could be established between the percentage of bilateral activity (both limbs active) and the levels of dopamine, noradrenaline and serotonin. In contrast, a negative correlation was found between the levels of noradrenaline and the frequency of the step cycles (speed of locomotion).

## 3. Discussion

Here, we show that preventing the action of the MAOs and COMT is an efficient strategy to trigger episodes of air-stepping in the newborn rat from a low, poorly effective dose of L-DOPA (25 mg/kg). Associated neurochemical data additionally confirm the neuromodulatory role that catecholamines already play early after birth at the levels of the cervical and lumbar locomotor networks controlling the fore- and hindlimbs, respectively.

### 3.1. Manipulating the Enzymatic Pathways Operating Spinal Monoamines

Monoamine oxidases and catechol-o-methyl transferase are two enzyme families that work synergistically to produce homovanillic acid (HVA) from dopamine. Dopamine produces HVA via the intermediate production of DOPAC in the case where MAO acts before COMT, or via the intermediate production of 3-MT, when COMT acts before MAO. Here, the dosage of intermediate by-products allowed us to ascertain the efficacy of the MAO and COMT inhibitors. This is evidenced by the absence of DOPAC and 3-MT in the group of animals that received nialamide or tolcapone without L-DOPA. In such a situation, one would expect an increase in the level of dopamine and/or of the by-product produced from the untargeted enzymatic pathway. However, dopamine was no longer detected in the spinal cord of animals receiving tolcapone or nialamide. Instead, we found an increase in the endogenous L-DOPA tissue content in both situations. We do not have clear explanation for this paradoxical response, but we are considering the following arguments. 

First, while the blockade of the COMT by tolcapone could tentatively explain the raise in L-DOPA levels, it is of note that COMT is presumably not expressed by catecholaminergic neurons in adult rats [25]. This finding could therefore suggest that L-DOPA produced by the activity of tyrosine hydroxylase can be released by some neurons expressing tyrosine hydroxylase and uptake ectopically, or that COMT is expressed by neurons expressing tyrosine hydroxylase during development. Second, the increase in endogenous L-DOPA induced by nialamide alone suggests that the increase in catecholamines (dopamine and/or NA) induced by nialamide directly raise back the content of L-DOPA. It is of note that a recent computational study suggested that endogenous L-DOPA is in fact more regulated than previously thought in dopaminergic neurons [26]. Third, it is plausible that DA, DOPAC and L-DOPA spinal contents (without exogenous L-DOPA) correspond more to noradrenergic neurons than dopaminergic neurons. Indeed, the noradrenergic innervation is massive compared to the dopaminergic one in this region. The metabolic activity within noradrenergic neurons is likely different compared to dopaminergic neurons of the striatum. Finally, the increase in basal levels of L-DOPA induced by nialamide or tolcapone appears to be massive (from non-detected values to above 50 pg/mg of tissue). In reality, it is likely an overestimation because the limit of quantification of L-DOPA in that experiment, due to its time of elution at the end of the solvent front was very low (estimated to be 30–40 pg in a sample). The HPLC conditions were initially satisfying the measurement of tissue L-DOPA in response to L-DOPA injection (very high levels, even after the lower dose of L-DOPA; [19]) but not basal L-DOPA tissue content.

The inhibitory action of nialamide on MAOs could be essentially ascertained by the levels of 5-HT and its sub-product 5-HIAA, which respectively increased and decreased in the spinal cord. As expected, the levels of 5-HT and 5-HIAA were not affected in the group receiving the COMT inhibitor.

When exogenous L-DOPA is injected to animals, it induces tremendous increases of the neurochemical indexes associated with DA including L-DOPA, DA, DOPAC or 3-MT, and weak effects or no effect on NA and 5-HT tissue content [27]. Briefly, the accumulation of DA markers after L-DOPA injection involves other cellular systems than dopaminergic terminals [28,29], and the metabolism of L-DOPA or DA involves MAO and/or COMT from various systems, and at different efficacies. For instance, exogenous L-DOPA in the presence of the COMTi tolcapone apparently decreased or did not affect L-DOPA tissue content, with a modest increase in DA tissue content, but this combined treatment dramatically increased DOPAC tissue content. This result suggested that the blockade of COMT metabolic pathway boosted the catabolic pathway associated with MAO. Conversely, exogenous L-DOPA in the presence of nialamide increased dopamine, noradrenaline, and 5-HT levels and, as expected decreased DOPAC levels. In that case, the levels of 3-MT were increased which indicates that the activity of the COMT was preserved (or enhanced). The neurochemical picture is complicated if we look at the results of L-DOPA (dose response, and or L-DOPA plus COMTi or MAOi) on 5-HT (and 5-HIAA) or NA tissue content. With regard to the 5-HT system, the effects of exogenous L-DOPA are likely region-dependent in the CNS [30,31] and involves several mechanisms including the chase of 5-HT from vesicles of exocytosis by newly-synthesized DA [32]. Thus, even though the responses of exogenous L-DOPA on 5-HT are puzzling, we have to keep in mind that our measurements were all performed 30 min after L-DOPA injection, and, according to the dose, the expression of the various mechanisms triggered by L-DOPA on 5-HT terminals would be more or less present. In any case, our data indicate that at the dose of 100 mg/kg, tolcapone and nialamide are efficient and do not exert an unspecific crossed-action. 

### 3.2. Regional Differences in Spinal Monoamines

Comparisons of monoamine levels in the cervical and lumbar spinal cord in the different experimental groups highlighted a few regional differences (Table 1). While the levels of 5-HT measured in the cervical and lumbar cord were not significantly different, the levels of noradrenaline were in most cases significantly higher in the cervical than in the lumbar spinal cord. Basal levels of dopamine were very low, and globally, loading the system with L-DOPA alone or in combination to nialamide or tolcapone led to a similar accumulation of dopamine in both spinal cord regions. 

One hypothesis is that the present biochemical data reflect the different maturational states of the monoaminergic systems in the spinal cord [33]. Indeed, the serotonergic and noradrenergic descending pathways are the first to invade the spinal cord during development [34,35]. The descending dopaminergic pathways take place later on during the 3–4 post-natal weeks [20,36]. Thus, from the embryonic to the late post-natal stage, dopamine is likely to be primarily produced by the AADC expressed in cellular elements intrinsic of the spinal cord [21], noradrenergic neurons, or possibly in serotonergic terminals [37]. No clear evidence from the literature indicates that the monoaminergic systems operating in the cervical and lumbar neuronal networks become equivalent at the adult stage.

We found the cervical and lumbar monoamines contents were positively correlated in animals that received exogenous L-DOPA. Thus, despite maturational differences along the spinal cord early after birth, monoamine levels can be homogenously manipulated in the rostral and caudal spinal cord.

### 3.3. Monoamines Contribution to L-DOPA Pro-Locomotor Action

Exogenous L-DOPA has long been established to activate the spinal locomotor programs in several vertebrate species [5,6,13,14,15,16,17]. In the present work, L-DOPA induced a dose-dependent increase of the number of newborn rats exhibiting air-stepping from 25 to 100 mg/kg. This is consistent with our first evaluation showing that 100% of the cohort exhibited air-stepping at the dose of 50 mg/kg L-DOPA and for prolonged periods of time from 75 mg/kg [19]. A higher dose of L-DOPA (150 mg/kg) did not reveal additional effects compared to those already seen at 100 mg/kg except a slightly higher proportion of quadrupedal stepping and number of hindlimbs steps. Thus, the present work highlights that the maximal effects of L-DOPA on expression air-stepping are almost reached at 100 mg/kg. It is noteworthy that the animals did not receive the inhibitor of the peripheral AADC, which is supposed to potentiate by 5 to 10 the entry of L-DOPA in the CNS [1]. 

The role of monoamines in the effects of exogenous L-DOPA is usually unclear in acknowledging that all monoamines can contribute to locomotor activity in newborn rats [38,39,40]. The tissue content of L-DOPA is not predictive of the magnitude of the effect of L-DOPA. Indeed, the combination of tolcapone (100 mg/kg)/L-DOPA (25 mg/kg) produced a nearly maximal locomotor response but the increase in spinal L-DOPA obtained in this condition was lower compared to the other conditions inducing a high magnitude of locomotor response.

The pro-locomotor action of L-DOPA in newborn rats indeed requires its conversion into dopamine through the action of the AADC [41]. The raise of dopamine induced by L-DOPA is possibly one important factor but in the present work the magnitude of the spinal dopamine content also does not always reflect the behavioral outputs. Supporting this assumption, the raise in tissue dopamine induced by 25 mg/kg L-DOPA is already important, but air-stepping response was only rarely triggered [19]. Moreover, even though the combination of tolcapone (100 mg/kg)/L-DOPA (25 mg/kg) triggers almost a maximal locomotor response, the tissue content of dopamine was not dramatically enhanced when compared to L-DOPA (100 and 150 mg/kg) alone or the combination nialamide/L-DOPA (25 mg/kg). Thus, the spinal contents of L-DOPA and dopamine do not solely explain the magnitude of the behavioral response. As explained above, the tissue content for dopamine and L-DOPA has no functional values after exogenous L-DOPA injection because it also reflects the ectopic accumulation of dopamine and L-DOPA in the tissue, L-DOPA virtually entering all cells via the transporter of L-aromatic amino acids [1]. Other parameters would be more appropriate, such as the measurement of extracellular levels of dopamine coming from serotonergic terminals [27,32,37,42], but the inherent surgery is a strong technical issue in pups. 

The spinal content of noradrenaline obtained after L-DOPA is extremely complex to interpret. Indeed, exogenous L-DOPA does not impact, or only marginally, the tissue content of noradrenaline even if it is a metabolic precursor and despite the concomitant increase in dopamine tissue content. As explained above, however, the raise in dopamine tissue content induced by L-DOPA witnesses a high rate of decarboxylation of L-DOPA in several cellular species, and the contribution of noradrenergic terminals in this increase is probably marginal [20,43]. In addition, an excess of dopamine or other trace amines in the cytosol of noradrenergic terminals might induce a flush of noradrenaline away from the vesicle of exocytosis, thereby exposing noradrenaline to degrading enzymes [44]. We indeed report that the blockade of COMT or MAO raised the tissue content of noradrenaline following exogenous L-DOPA administration.

The results are also interesting as regards the role of 5-HT in the locomotion promoted by L-DOPA. Our results indicate that the increase in 5-HT tissue content when nialamide and L-DOPA are combined positively acts on the locomotor activities of the pups. In our recent publication the co-administration of 5-HTP deteriorated the pro-locomotor action induced by L-DOPA [19]. This effect was associated with a logical increase of 5-HT tissue content occurring at the expense of the increase of DA tissue content produced by L-DOPA. The data obtained with nialamide would favor the hypothesis that the deterioration of L-DOPA-induced air-stepping by 5-HTP is not related to 5-HT itself. Instead, the negative effect of 5-HTP would be related to the direct impairment of the decarboxylation of L-DOPA into dopamine and/or the co-storage of dopamine and 5-HT in vesicles of exocytosis of 5-HT terminals [32,45,46].

### 3.4. Monoaminergic Control of Locomotor Network Excitability

Previous works have shown that blocking dopaminergic or noradrenergic signaling suppresses the capacity of L-DOPA to trigger episodes of air-stepping in newborn rats [47,48,49]. On the other hand, activating the serotonergic system with quipazine alone or in combination with L-DOPA is also a strategy that can be used to activate air-stepping in intact and spinal cord injured newborn rats [50]. These data not only indicate that all three monoaminergic systems may contribute to the expression of air-stepping, but also that they can directly activate the cervical and lumbar spinal locomotor networks controlling the forelimb and hindlimb, respectively [51,52]. In this context, we found here a positive correlation between the levels of each of the 3 monoamines in the spinal cord and the amount of locomotor activity.

Here, robust episodes of air-stepping were triggered by either moderate/high doses L-DOPA alone, or by combining a lower dose of L-DOPA with nialamide or tolcapone. None of these pharmacological strategies led to equivalent levels of all three monoamines in the spinal cord. High doses of L-DOPA only increased dopamine, serotonin and noradrenaline levels being unaffected. In the low L-DOPA/tolcapone condition, dopamine is less increased, serotonin level remains similar, but noradrenaline level is increased. Finally, low L-DOPA/nialamide is the condition that led to the highest levels of monoamines in the spinal cord. These different pharmacological conditions set different biochemical states and consequently lead to different neuromodulatory contexts. The fact that air-stepping was consistently obtained in these different conditions is highly consistent with the role of neuromodulation in the regulation of rhythmic neural networks operations [53,54]. In the context of locomotion, different neuromodulators or combination of neuromodulators, can activate the spinal locomotor programs in newborn rats and mice [38,39,40,55,56,57,58,59]. Recent studies have indicated that neuromodulators such as dopamine and serotonin can actually tune the excitability state of the spinal networks up to an activation of the locomotor programs [60,61]. Here, we confirm that different pharmacological strategies differentially manipulating the monoaminergic system in vivo can be used to reach a locomotor state. A detailed analysis of the locomotor pattern revealed that the operation of the locomotor network still remained flexible as a function of the neuromodulatory context. For instance, although the spinal content of dopamine does not interact with the locomotor frequency [19], our correlative analyses show a negative correlation between noradrenaline levels and the frequency of the locomotor movements. Thus, monoamines not only contribute to reach a locomotor state in vivo but are likely also to finely tune the operation of the locomotor circuits.

## 4. Materials and Methods

Experiments were performed at post-natal day 5 (P5) on Sprague–Dawley newborn rats (*n* = 159) of either sex. The rats were bred in the laboratory. This specific developmental stage has been chosen based on previous works [17,18,19,41,47,49]. All procedures followed the European Committee Council directives (2010/63/EU) and were conducted in accordance with the local ethics committee of the University of Bordeaux, and validated by the French Ministry for Higher Education, Research and Innovation (APAFIS #11978). All efforts were made to minimize animal suffering and the number of animals used. Each animal was subjected to only one of the experimental pharmacological conditions.

Drugs. L-3,4-dihydroxyphenylalanine (L-DOPA, ab120573, Abcam Biochemicals, Cambridge, UK), nialamide (252999, Sigma Life Science - Merck, Darmstadt, Germany) and tolcapone (SML0150, Sigma Life Science - Merck, Darmstadt, Germany), all in the base form, were freshly prepared on the day of the experiment. L-DOPA (50 mg) was dissolved in HCl (37%, 350 µL), distilled water (150 µL) and Na2HPO4 (4.5 mL), and stirred to obtain a clear homogenous solution at a concentration of 10 mg/mL. This vehicle solution was administrated without L-DOPA in the vehicle group, and in the groups receiving nialamide or tolcapone alone (see Figure 1). Nialamide and tolcapone were dissolved in NaCl (0.9%) at the same concentration of 10 mg/mL. Drugs were administered subcutaneously in the nape using a 30 G needle. L-DOPA and vehicle solution were injected in a volume of 0.15 mL/10 g body weight. Nialamide and tolcapone were injected in a volume of 0.1 mL/10 g body weight.

Air-stepping. The methodology is similar to one described in a previous work [19]. Adhesive hemi-spherical makers (diam 3 mm, optitrack, NaturalPoint, Corvallis, OR, USA) were positioned on the hip, scapula and toes of the fore- and hindlimbs bilaterally. Following L-DOPA administration, animals were placed in a sling suspended in the air. Air-stepping activities were investigated from 27 to 30 min after drug injection at which the L-DOPA effect is maximal [18]. Animals were video monitored using either a pair of synchronized cameras controlled by two Raspberry Pi microcomputers (https://www.raspberrypi.org) or a pair of cameras (Basler) controlled and synchronized using StreamPix acquisition software (StreamPix 6, NorPix, Montreal, QC, Canada). The frame rate was set to 49 images per second and video recordings were stored on a server for off-line analyses. The x-y coordinates of the kinematic markers were extracted using DeepLabCut [62], allowing for the calculation of the angular excursion of each limb using Matlab routines (Mathworks, Natick, MA, USA). Angular excursions of the pendular movements of the forelimb were calculated from the angles formed between the toe, scapula and hip, and angular excursions of hindlimb from the angle formed between the toe, hip and scapula. The locomotor cycle period was calculated as the time interval between two successive protracted positions of the limb (i.e., time for a backward retraction plus subsequent forward protraction). The amplitude of pendular movements was defined as the angles formed between the extreme forward and backward leg positions during a step cycle, which are analogous to the swing and stance phases, respectively, during overground locomotion. The phase relationships between pairs of limbs were calculated from the point of swing onsets. For a given pair of limbs, the phase was derived from the delay between the swing start of the reference limb and the next swing start of the compared limb divided by the period of the reference limb step cycle. The phase relationship was converted into radians for circular statistical analysis and illustrated as circular plots. On circular plots, the directions were normalized between 0 and 1, with the direction 0/1 indicating a synchrony of the locomotor movements between the considered pair of limbs (such as during bound), 0.5 an alternation of the locomotor movements (such as during normal walking) and values around 0.25 or 0.75 representing out-of-phase locomotor movements (indicating a shift in locomotor movements corresponding to 25% or 75% of the step cycle of the limb taken as reference [63]).

Spinal cord samples. Next, 30 min after L-DOPA injection, animals were sacrificed by decapitation. The spinal cord was rapidly extracted under a binocular magnifier using surgical forceps and micro-scissors. The cervical and lumbar enlargements were immediately frozen in isopentane (−34 °C) and stored at −80 °C in Eppendorf tubes. These spinal tissues were later used to dose the monoamines levels by HPLC (see below).

Quantification of monoamine levels. Monoamines levels were measured in the spinal cord via HPLC following the protocol we described recently [19,64]. At the time of biochemical analysis, the tubes containing the samples of spinal cord were placed on ice, and rapidly wiped and weighed again in order to get the weight of the spinal samples. Samples were next homogenized in 0.1 N HClO4 (100 µL), sonicated and centrifuged at about 16,000× *g* (13,000 rpm, Eppendorf Centrifuge 5424R, rotor type FA-45-24-11) for 30 min at 4 °C. The supernatants were injected into the HPLC system using a manual injector (Rheodyne 7725i, C.I.L. Cluzeau, Sainte-Foy-La-Grande, France) equipped with a 20 µL loop into the HPLC column (Hypersyl C18, 150 × 4.6 mm, 5 µm, C.I.L. Cluzeau, Sainte-Foy-La-Grande, France) preceded by a Brownlee–Newgard precolumn (RP-8, 15 × 32 mm, 7 µm, C.I.L. Cluzeau, Sainte-Foy-La-Grande, France). The filtered (0.22 mm Millipore filter) mobile phase was composed of (in mM): 60 NaH2PO4, 0.1 Disodium EDTA, 2-Octane-Sulfonic acid in deionized water (18 MW cm^2^) containing 7% methanol. The mobile phase was delivered by an HPLC pump (LC-20-AD, Shimadzu France, Marne-la-Vallée, France) at 1.2 mL/min. The pH was adjusted to 4 with orthophosphoric acid to obtain good conditions of eluent separation in the chromatogram. Monoamine detection was obtained with a coulometric cell (5011 coulometric cell, ESA, Paris, France) coupled to a programmable detector (Coulochem II, ESA, Chelmsford, MA, USA). The potential of the electrodes was set to −270 and +350 mV. At these potentials (mainly oxidation), several metabolites and L-DOPA-derived metabolites can be oxidized which can lead to confounding pics [44]. Thus, we paid attention to the fact that the elution time for adrenaline, 5-hydroxyindolacetic acid, 3,4-dihydroxyphenylacetic acid, 3-O-methyl-DOPA, vanylmandelic acid, homovanillic acid, 3-methoxytyramine or 5-hydroxytryptophan was separated from the elution time for the compounds of interest. Moreover, we also confirmed that DOPEG, MOPEG, octopamine, tyramine, tyrosine and tryptophan induced poor or undetectable signals at these potentials [65]. Signals were acquired using an Ulys2 interface (Datalys, Saint-Martin-d’Hères, France) and stored on a computer using dedicated software (Azur 5.0, Datalys, Saint-Martin-d’Hères, France). The time for a sample elution was approximately 32 min. Calibration curves were obtained using standard solutions at three distinct and known concentrations for each compound of interest. Moreover, a standard solution (1 ng/10µL) was administered before and after each daily series of sample analysis. The limit of detection of the different compounds was (in pg/10 µL): L-DOPA, 5 pg; NA, 4 pg; DOPAC, 2 pg; DA, 2 pg; 5-HIAA, 4 pg; 5-HT, 12 pg; 3-MT, 20 pg. Of note, the limit of quantification of L-DOPA was bad in the context of the samples because L-DOPA was eluted at the end of the solvent front, which was large in these samples implying its detection only above 30 pg in the sample.

Pharmacological treatment and experimental design. The experiments were conceived to determine the extent to which tolcapone (30 and 100 mg/kg) or nialamide (100 mg/kg) could boost the behavioral effects triggered by sub-optimal dose of L-DOPA. Based on our previous investigation [19], we decided first to study the response of L-DOPA at 25 mg/kg (expecting low rate of responses), 100 mg/kg (expecting high rate of responses), and 150 mg/kg to approximate a plausible, maximal rate of responses. On the basis of this dose response, we studied the effect of the combination of L-DOPA (25 mg/kg) with nialamide (100 mg/kg) or tolcapone (30 or 100 mg/kg) on episodes of air-stepping and compared the resulting effects with the dose response. Based on previous data in the literature [7,66,67], nialamide and tolcapone were injected 120 and 60 min before L-DOPA (or its vehicle) corresponding to 150 and 90 min before the sacrifice to ensure the inhibition of the MAOs or the COMT, respectively.

Statistics. Neurochemical data were analyzed using Prism software (Prism9, GraphPad software, Boston, MA, USA). Descriptive statistics are presented as mean ± standard deviation (SD), or as median/IQR (interquartile range) for parametric and non-parametric data sets, respectively. Comparisons between groups were achieved with a confidence level set at 95% using the tests mentioned in the text and tables. Correlations of Pearson between biochemical data and locomotor parameters were assessed taking parameters two by two [24]. This results in the calculation of a correlation coefficient r, which quantify the direction (positive or negative) and strength of the correlation, and that was considered significant for a *p* value < 0.05. Circular statistics were performed on raw data using the circular statistic toolbox for Matlab [63]. Circular data are represented as polar plots on which the direction of the resultant vector r indicates the mean angular value (normalized from 0 to 1), while the length of the vector is a measure of the concentration of the data around that mean. The non-uniform distribution of the data was assessed using the Raleigh test with a significance level set at *p* < 0.05. Only data showing a non-uniform distribution were represented as polar plots. Inter-groups comparisons of circular data were performed using the Watson–Williams test. 

## 5. Conclusions

Investigating the development of locomotor functions in newborn animals allows for the understanding of the maturation of the descending neuromodulatory regulation of spinal sensorimotor networks. Here, we combined behavioral and biochemical approaches to unravel how a pharmacological strategy impact the neuromodulatory substrates onto which a specific behavior—i.e., air-stepping movements—may emerge or not. Focusing primarily on the spinal cord component where the networks organizing the locomotor movements of the limbs are located, our study calls for additional analyses aiming at unraveling the contribution of the supraspinal biochemical mechanisms in the regulation of air-stepping activity in newborn rats.

## Figures and Tables

**Table 1:** Spinal monoamines levels in the different pharmacological conditions. Statistical differences between the cervical and lumbar spinal cord are indicated in bold together with the associated *p*-value. N/A not available (not detected).

	L-DOPA		Wilcoxon *p* value	Dopamine		Wilcoxon *p* value
	Cervical	Lumbar		Cervical	Lumbar	
**Saline**	N/A	N/A	N/A	18 (12/23)	12 (7/15)	*0.0645*
**Nial.** 100 mg/kg	76 (69/98)	70 (62/126)	*0.4316*	N/A	N/A	N/A
**Tolc.** 100 mg/kg	79 (70/119)	108 (76/126)	*0.5566*	N/A	N/A	N/A
**L-DOPA** 25 mg/kg	1486 (1262/2137)	1458 (1066/2186)	*0.0955*	**546 (421/677)**	**411 (345/694)**	***0.0047***
100 mg/kg	**6740 (6310/8531)**	**5523 (5117/7239)**	***0.0018***	2516 (2119/2975)	2623 (1994/3151)	*0.8532*
150 mg/kg	**9812 (8358/10516)**	**7035 (6161/8837)**	***0.0005***	2982 (2546/3640)	2757 (2372/3208)	*0.0522*
**L-DOPA 25 mg/kg**						
**+ Tolc** 30 mg/kg	**638 (552/830)**	**1793 (1514/2325)**	***<0.0001***	638 (552/830)	631 (525/756)	*0.6333*
**+ Tolc** 100 mg/kg	**1056 (962/1329)**	**3512 (3022/3997)**	***0.0005***	**1056 (962/1329)**	**1251 (1126/1883)**	***0.0269***
**+ Nial** 100 mg/kg	**3750 (2987/4918)**	**2510 (1726/3323)**	***0.0039***	3750 (2987/4918)	3731 (2892/4189)	*0.3223*
	**DOPAC**		Wilcoxon *p* value	**3-MT**		Wilcoxon *p* value
	Cervical	Lumbar		Cervical	Lumbar	
**Saline**	12 (6/15)	9 (4/12)	*0.3013*	71 (37/94)	N/A	N/A
**Nial.** 100 mg/kg	N/A	N/A	N/A	N/A	N/A	N/A
**Tolc.** 100 mg/kg	N/A	N/A	N/A	N/A	N/A	N/A
**L-DOPA** 25 mg/kg	**1425 (1079/2038)**	**1296 (833/1656)**	***0.0400***	50 (32/137))	46 (37/52)	*0.1230*
100 mg/kg	**3651 (2950/4325)**	**3074 (2472/4080)**	***0.0269***	108 (91/142)	102 (79/126)	*0.0650*
150 mg/kg	**5497 (4385/6302)**	**4499 (3778/4846)**	***0.0010***	N/A	N/A	N/A
**L-DOPA 25 mg/kg**						
**+ Tolc** 30 mg/kg	2930 (2580/3874)	2973 (2531/3804)	*0.4980*	N/A	N/A	N/A
**+ Tolc** 100 mg/kg	4220 (3589/4516)	4417 (3926/5055)	*0.3394*	N/A	N/A	N/A
**+ Nial** 100 mg/kg	472 (293/684)	536 (350/673)	*0.2163*	1888 (1449/2749)	1747 (1230/2384)	*0.0522*
	**Noradrenaline**		Wilcoxon *p* value	**Serotonin**		Wilcoxon *p* value
	Cervical	Lumbar		Cervical	Lumbar	
**Saline**	**206 (184/240)**	**158 (132/176)**	***0.0269***	264 (237/284)	243 (226/296)	*0.3203*
**Nial.** 100 mg/kg	**255 (204/263)**	**195 (148/221)**	***0.0195***	306 (224/337)	321 (214/398)	*0.4316*
**Tolc.** 100 mg/kg	196 (184/210)	182 (147/210)	*0.1602*	174 (142/191)	198 (190/215)	*0.1309*
**L-DOPA** 25 mg/kg	**262 (184/291)**	**161 (121/259)**	***0.0002***	192 (139/274)	228 (183/251)	*0.1688*
100 mg/kg	**215 (175/251)**	**172 (146/193)**	***<0.0001***	258 (195/328)	239 (200/322)	*0.7915*
150 mg/kg	**312 (279/371)**	**189 (167/238)**	***0.0005***	146 (122/204)	129 (98/186)	*0.3013*
**L-DOPA 25 mg/kg**						
**+ Tolc** 30 mg/kg	**355 (331/392)**	**316 (277/336)**	***0.0012***	158 (115/222)	168 (130/226)	*0.1281*
**+ Tolc** 100 mg/kg	367 (305/414)	339 (321/364)	*0.3804*	254 (207/299)	269 (238/330)	*0.3804*
**+ Nial** 100 mg/kg	**387 (312/420)**	**273 (229/304)**	***0.0020***	528 (412/586)	458 (404/567)	*0.6250*
	**5HIAA**		Wilcoxon *p* value			
	Cervical	Lumbar				
**Saline**	101 (79/120)	126 (68/147)	*0.4143*			
**Nial.** 100 mg/kg	**59 (49/67)**	**94 (51/106)**	***0.0391***			
**Tolc.** 100 mg/kg	80 (72/102)	114 (89/129)	*0.0977*			
**L-DOPA** 25 mg/kg	90 (74/137)	115 (98/128)	*0.0799*			
100 mg/kg	**172 (146/198)**	**217 (171/230)**	***0.0003***			
150 mg/kg	114 (83/128)	122 (113/168)	*0.0522*			
**L-DOPA 25 mg/kg**						
**+ Tolc** 30 mg/kg	**78 (53/100)**	**85 (69/103)**	***0.0002***			
**+ Tolc** 100 mg/kg	**117 (92/138)**	**141 (119/184)**	***0.0122***			
**+ Nial** 100 mg/kg	**102 (85/146)**	**160 (103/262)**	***0.0210***			

**Table 2:** Comparison of monoamines levels in the cervical and lumbar spinal cord between the group receiving L-DOPA at different doses. Statistical differences are indicated in bold together with the associated *p*-value. ↗ increase, ↘ decrease.

	L-DOPA				Dopamine			
	Cervical		Lumbar		Cervical		Lumbar	
**Kruskal-Wallis test**	*p* Value		*p* Value		*p* Value		*p* Value	
	***<0.0001***		***<0.0001***		***<0.0001***		***<0.0001***	
**Dunn’s multiple** **comparisons**		*p* Value		*p* Value		*p* Value		*p* Value
L-DOPA 25 vs. L-DOPA 100	↗	***<0.0001***	↗	***<0.0001***	↗	***<0.0001***	↗	***<0.0001***
L-DOPA 25 vs. L-DOPA 150	↗	***<0.0001***	↗	***<0.0001***	↗	***<0.0001***	↗	***<0.0001***
L-DOPA 100 vs. L-DOPA 150		*0.1102*		*0.5045*		*0.6345*		*>0.9999*
	**DOPAC**				**Noradrenaline**			
	**Cervical**		**Lumbar**		**Cervical**		**Lumbar**	
**Kruskal-Wallis test**	*p* Value		*p* Value		*p* Value		*p* Value	
	***<0.0001***		***<0.0001***		***0.0005***		*0.2799*	
**Dunn’s multiple** **comparisons**		*p* Value		*p* Value		*p* Value		*p* Value
L-DOPA 25 vs. L-DOPA 100	↗	***<0.0001***	↗	***<0.0001***		*0.2692*		
L-DOPA 25 vs. L-DOPA 150	↗	***<0.0001***	↗	***<0.0001***		*0.0601*		
L-DOPA 100 vs. L-DOPA 150	↗	***0.0280***		*0.1103*	↗	***0.0003***		
	**5-HT**				**5-HIAA**			
	**Cervical**		**Lumbar**		**Cervical**		**Lumbar**	
**Kruskal-Wallis test**	*p* Value		*p* Value		*p* Value		*p* Value	
	***0.0030***		***0.0001***		***<0.0001***		***<0.0001***	
**Dunn’s multiple** **comparisons**		*p* Value		*p* Value		*p* Value		*p* Value
L-DOPA 25 vs. L-DOPA 100		*0.1278*		*0.3816*	↗	***<0.0001***	↗	***<0.0001***
L-DOPA 25 vs. L-DOPA 150		*0.4286*	↘	***0.0151***		*>0.9999*		*>0.9999*
L-DOPA 100 vs. L-DOPA 150	↘	***0.0029***	↘	***<0.0001***	↘	*0.0052*	↘	*0.0018*

**Table 3:** Comparison of monoamines levels in the cervical and lumbar spinal cord between the groups receiving L-DOPA alone or combined with nialamide or tolcapone. The group L-DOPA 25 serves as reference for the multiple comparison.

	L-DOPA				Dopamine			
	Cervical		Lumbar		Cervical		Lumbar	
**Kruskal-Wallis test**	*p* Value		*p* Value		*p* Value		*p* Value	
	***<0.0001***		***<0.0001***		***<0.0001***		***<0.0001***	
**Dunn’s multiple** **comparisons**		*p* Value		*p* Value		*p* Value		*p* Value
L-DOPA 25 vs.								
L-DOPA 25 + Tolc. 30	↘	***<0.0001***		*0.4001*		*0.8183*		*0.3636*
L-DOPA 25 + Tolc. 100		*0.4795*	↗	***<0.0001***	↗	***0.0005***	↗	***<0.0001***
L-DOPA 25 + Nial. 100		*0.0574*	↗	***0.0182***	↗	***<0.0001***	↗	***<0.0001***
	**DOPAC**				**Noradrenaline**			
	**Cervical**		**Lumbar**		**Cervical**		**Lumbar**	
**Kruskal-Wallis test**	*p* Value		*p* Value		*p* Value		*p* Value	
	**<0.0001**		**<0.0001**		**0.0004**		**<0.0001**	
**Dunn’s multiple** **comparisons**		*p* Value		*p* Value		*p* Value		*p* Value
L-DOPA 25 vs.								
L-DOPA 25 + Tolc. 30	↗	***0.0010***	↗	***0.0009***	↗	***0.0022***	↗	***0.0007***
L-DOPA 25 + Tolc. 100	↗	***<0.0001***	↗	***<0.0001***	↗	***0.0038***	↗	***<0.0001***
L-DOPA 25 + Nial. 100	↘	***0.0377***		*0.1099*	↗	***0.0021***		*0.6740*
	**5-HT**				**5-HIAA**			
	**Cervical**		**Lumbar**		**Cervical**		**Lumbar**	
**Kruskal-Wallis test**	*p* Value		*p* Value		*p* Value		*p* Value	
	***<0.0001***		***<0.0001***		***0.0104***		***<0.0001***	
**Dunn’s multiple** **comparisons**		*p* Value		*p* Value		*p* Value		*p* Value
L-DOPA 25 vs.								
L-DOPA 25 + Tolc. 30		*>0.9999*		*0.6024*		*0.1986*	↘	*0.0366*
L-DOPA 25 + Tolc. 100		*0.3188*		*0.2760*		*0.4940*		*0.2319*
L-DOPA 25 + Nial. 100	↗	***<0.0001***	↗	***<0.0001***		*>0.9999*		*0.2503*

## Data Availability

All data supporting the results of this study are in the manuscript, figures and tables.
